# Editorial: Plant programmed cell death revisited, volume II

**DOI:** 10.3389/fpls.2023.1281902

**Published:** 2023-09-07

**Authors:** Joanna Kacprzyk, Arunika H. L. A. N. Gunawardena, Francois Bouteau, Paul F. McCabe

**Affiliations:** ^1^ School of Biology and Environmental Science, University College Dublin, Dublin, Ireland; ^2^ Biology Department, Faculty of Science, Dalhousie University, Halifax, NS, Canada; ^3^ Laboratoire Interdisciplinaire des Énergies de Demain, Université de Paris, Paris, France

**Keywords:** plant programmed cell death, aerenchyma, genome wide association study, ferroptotic cell death, mitochondria, biotic interactions, seedless phenotype, panicle abortion

Programmed cell death (PCD) is a fundamental process in plants, involved in plant development and mediating abiotic and biotic environmental interactions (Kacprzyk et al., 2011; Locato and De Gara, 2018). While our understanding of genetic regulation of plant PCD, and the sequence of molecular events involved, is still relatively fragmented, the potential of PCD research to inform development of future-proofed crop cultivars is increasingly recognized. This Research Topic compiles valuable contributions that highlight the increasing momentum of this research area, and reports the use of a diversity of approaches for studying cell death in plants.

The seven Original Research Articles comprising the *Plant Programmed Cell Death Revisited, Volume II* explore plant PCD in the context of development (Hu et al., Htwe et al., Xie et al.), plant-pathogen interactions (Dong et al., Nguyen et al.) and responses to environmental pollution (Leppälä et al.); in addition to providing insights into PCD-associated proteomic changes associated occurring within mitochondria and cytosol (Schwarze et al.). These studies were conducted using a diverse array of experimental systems and species, from cultured *Arabidopsis thaliana* cells to lotus (*Nelumbo nucifera*) roots and fruit of oil palm (*Elaeis guineensis* Jacq.), while utilising a wide range of methodologies including transcriptomics, proteomics and genome wide association studies (GWAS).

Htwe et al. provided new data suggesting that PCD leads to pollen tube lethality, resulting in double fertilization failure and seedless phenotype in oil palm. Seedless fruits are a desirable phenotype in oil palm, as they have higher oil content thanks to elimination of the kernel and an increase in the mesocarp volume, leading to lower processing costs. The transcriptome analyses suggested that the seedless phenotype might be driven by expression of several PCD-related genes; including S-RNases, that are known to be involved in PCD occurring during self-incompatibility in other species (Hua et al., 2008; Xie et al., 2022a). Excitingly, the authors developed two S-RNase based simple sequence repeats (SSR) markers for identification of the seedless phenotype which give their work potential to lead to increased yields of palm oil production. Hu et al. identified the decreased levels of a proton pump, plasma membrane localized, ATPase protein as the underlying cause of the phenotype observed in *lmpa* (lesion mimic leaf and panicle apical abortion) rice mutant. The *lmpa* plants also demonstrate increased sensitivity to heat and salinity, and the authors propose that loss of reactive oxygen species (ROS) homeostasis, due to disrupted intracellular and extracellular proton gradient, is responsible for the PCD-mediated panicle degeneration and lesion mimic formation. As rice yields are related to panicle architecture (Yang et al., 2021), these findings are highly relevant from the point of view of food security. The role of PCD in the context of plant development was also investigated in the Topic by Xie et al. who studied development of aerenchyma in lotus, an important aquatic economic crop with high edible, medicinal, ornamental, and ecological restoration value. Aerenchyma is a tissue that allows gas diffusion between roots and shoots leading to alleviation of potential hypoxia-induced damage caused by the water environment (Gunawardena et al., 2001; Evans, 2004). The authors tracked the cytological and biochemical features of PCD occurring during aerenchyma formation in *N. nucifera* roots and demonstrated that the process is promoted by ethylene signaling. The results suggested that aerenchyma formed in *N. nucifiera* belongs to mixed, schizo-lysigenous category and involves both cell separation and cell death.

In two other articles from the Topic, Dong et al. and Nguyen et al. investigated PCD events mediating plant-pathogen interactions. Dong et al. isolated 200 putative proteins secreted from salivary glands of *Riptorus pedestris*, a hemipteran pest that reduces yields of leguminous plants such as soybean. The transient expression assays in *N. benthamiana* allowed identification of effectors inducing bursts of ROS and local lesions formation at the infestation sites, this way manipulating plant immunity and facilitating insect feeding. The study provides novel insights into the mechanisms underlying crop yield losses caused by *R. pedestris* and will inform future strategies for control of this agricultural pest species. Nguyen et al. investigated ferroptotic cell death and defense response in rice during infection with the blast fungus *Magnaporthe oryzae*. The authors characterized rice ferritin 2, OsFER2, as a positive regulator of iron- and ROS-dependent ferroptotic cell death and immune response in avirulent *M. oryzae* interactions and proposed an elegant model for associated signaling pathway. These are critical findings in the context of food security, given that *M. oryzae* is responsible for approximately 30% of rice production losses globally, the equivalent of feeding 60 million people (Nalley et al., 2016). The regulation of plant PCD induced by abiotic stress was explored in this Topic by Leppälä et al. who advanced the understanding of cell death responses induced by harmful gaseous environmental pollutants: ozone (O_3_) and nitrogen dioxide (NO_2_) via combination of transcriptomic analyses and GWAS. The O_3_ and NO_2_ demonstrated largely overlapping transcriptional responses, but some differences were also identified by the authors, for example in terms of contrasting transcriptional regulation of RBOHF (RESPIRATORY BURST OXIDASE PROTEIN F) that suggested distinct signaling pathways activated by both gases. Furthermore, analysis of O_3_ and NO_2_ tolerance in natural Arabidopsis accessions allowed identification of several putative regulators of oxidative and nitrosative stress induced cell death, including ABH1(ABSCISIC ACID HYPERSENSITIVE 1/CAP-BINDING PROTEIN 80), that functions in abscisic acid signaling, mRNA splicing and miRNA processing. The generated data will benefit future research on how plants can combat the negative effects of air pollution. Finally, new molecular insights into events associated with early stages of plant PCD were provided by Schwarze et al., who combined a well-established system for studying PCD, *Arabidopsis thaliana* cell suspension culture, with subcellular fractionation and mass-spectrometry proteome profiling. The *A. thaliana* suspension cultures enabled induction and precise monitoring of PCD rates, as well as chemical manipulation of this process. The study underscored the importance of proteasome function, chaperone-mediated protein folding, and heat shock proteins (HSPs) in life-death decision in plant cells, and identified 113 proteins that may undergo early, controlled release from plant mitochondria following PCD-inducing treatments. The data generated by Schwarze et al. will be of relevance for research efforts aimed at further dissecting the role of mitochondrion in early plant PCD signaling pathways.

We hope that the broad plant development and stress responses community will find the new results reported within this Topic both useful and interesting. This Research Topic yet again highlights how ubiquitous PCD is in plant processes; and underlines the potential of PCD research to contribute to solutions for the key challenges that need to be addressed to maintain plant growth and production in the future.

## Author contributions

JK: Writing – original draft, Writing – review & editing. AG: Writing – review & editing. FB: Writing – review & editing. PM: Writing – review & editing.
